# Dramatic response of aneurysmal bone cyst to denosumab: Case report and literature review

**DOI:** 10.1002/ccr3.4993

**Published:** 2021-10-26

**Authors:** Pedram Fadavi, Amir Mohammad Arefpour, Ramyar Hariri, Maryam Vasheghani, Maryam Garousi, Farzad Taghizadeh‐Hesary

**Affiliations:** ^1^ Radiation Oncology Department Iran University of Medical Science Tehran Iran; ^2^ Radiation Oncology Department Shahid Beheshti University of Medical Science Tehran Iran

**Keywords:** aneurysmal bone cyst, denosumab, management, RANK ligand, resistant

## Abstract

Denosumab, a monoclonal antibody that specifically targets cytokine receptor activator of nuclear factor‐kappa B ligand (RANKL), is a potentially viable option in resistant aneurysmal bone cysts.

## Graphical abstract

Denosumab, a monoclonal antibody that specifically targets cytokine receptor activator of the nuclear factor‐kappa B ligand (RANKL), is a potentially viable option in resistant aneurysmal bone cysts.

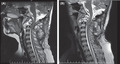

## 1 INTRODUCTION

Aneurysmal bone cysts (ABCs) are rare benign but locally aggressive lesions. Surgery and embolization are commonly the first‐line options; however, some cases are unresponsive. There is encouraging evidence for using denosumab in the second line. This work aims to report an unresponsive ABC case treated well by denosumab.

Aneurysmal bone cysts (ABCs) are infrequent, benign tumor‐like lesions involving axial and appendicular bones. Approximately 85% of ABCs occur in the second decade of life, although it might affect any age group. ABC usually affects long bones, and the spine (vertebral bodies) is the second affected site. Although biologically benign, ABCs tend to grow aggressively, causing adjacent bone and soft tissue destruction.^1^ ABCs are composed of two groups of cells: (i) osteoclast‐like multinucleated giant cells expressing receptor activator of nuclear kappa B (RANK) receptors, and (ii) neoplastic stromal cells expressing the RANK ligand (RANKL). The upregulated RANK–RANKL signaling axis can promote osteoclast‐dependent bone resorption in ABC, similar to giant cell tumor of bone (GCTB).^2^

Due to the rarity of ABC, the optimal treatment choice remains a matter of debate. Surgery (surgical resection or intralesional curettage), radiation therapy, sclerotherapy, selective arterial embolization, and intralesional injections (e.g., calcitonin and methylprednisolone) are among the treatment strategies.^2^, ^3^ The successful results of denosumab in the treatment of GCTB have persuaded clinicians to apply this strategy in treating the ABC. Denosumab, a human monoclonal antibody binding to RANKL, has been FDA approved for (i) prevention of skeletal‐related events in multiple myeloma and solid tumors, (ii) refractory malignancy‐induced hypercalcemia, (iii) unresectable GCTB in skeletally mature individuals, (iv) bone loss in breast and prostate cancer, and (v) glucocorticoid‐induced osteoporosis.^4^ However, denosumab is not yet FDA‐approved for ABC because of its limited existing literature.^2^

We report a challenging ABC case in the cervical spine, resistant to surgery, selective arterial embolization, and radiation therapy, with significant regression to the first dose of denosumab. In addition, we review the relevant literature.

## 2 CASE HISTORY/EXAMINATION

A previously healthy 13‐year‐old boy presented with a three‐month history of neck pain, swelling, stiffness, and movement restriction in his neck without neurologic dysfunction in December 2019. He had a good general condition (Eastern Cooperative Oncology Group [ECOG] = 0), with normal neurologic examination.

## 3 DIFFERENTIAL DIAGNOSIS, INVESTIGATIONS, AND TREATMENT

**FIGURE 1:**
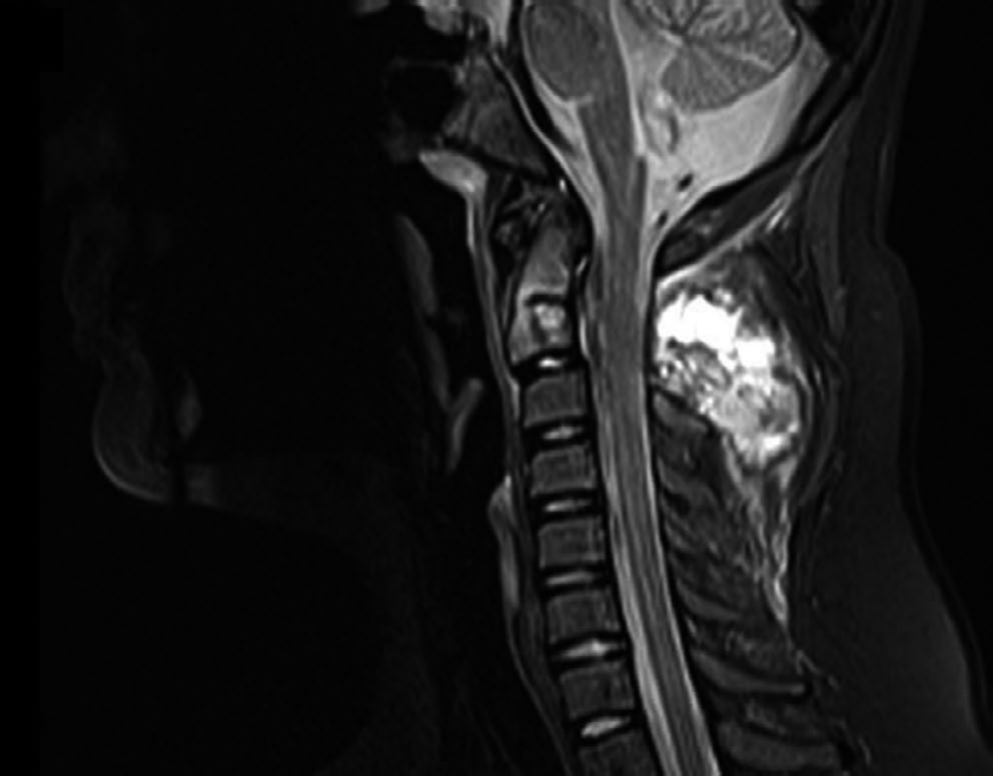
Craniocervical T2W sagittal image in favor of aneurysmal bone cyst at the level of C2

The cervical computed tomography (CT) scan showed a large mass (66 × 55 × 46 mm) with calcification and lytic changes at the level of C2. The cervical magnetic resonance imaging (MRI) showed a large mass containing a characteristic fluid–fluid level and hemorrhage in the C2 spinous process extending to C2 pedicles and thick bulging of the mass to the right side of the spinal canal, causing pressure on the thecal sac (Figure 1). MR findings raised the following differential diagnoses: (i) ABC, (ii) GCTB, and (iii) telangiectatic osteosarcoma.

**FIGURE 2:**
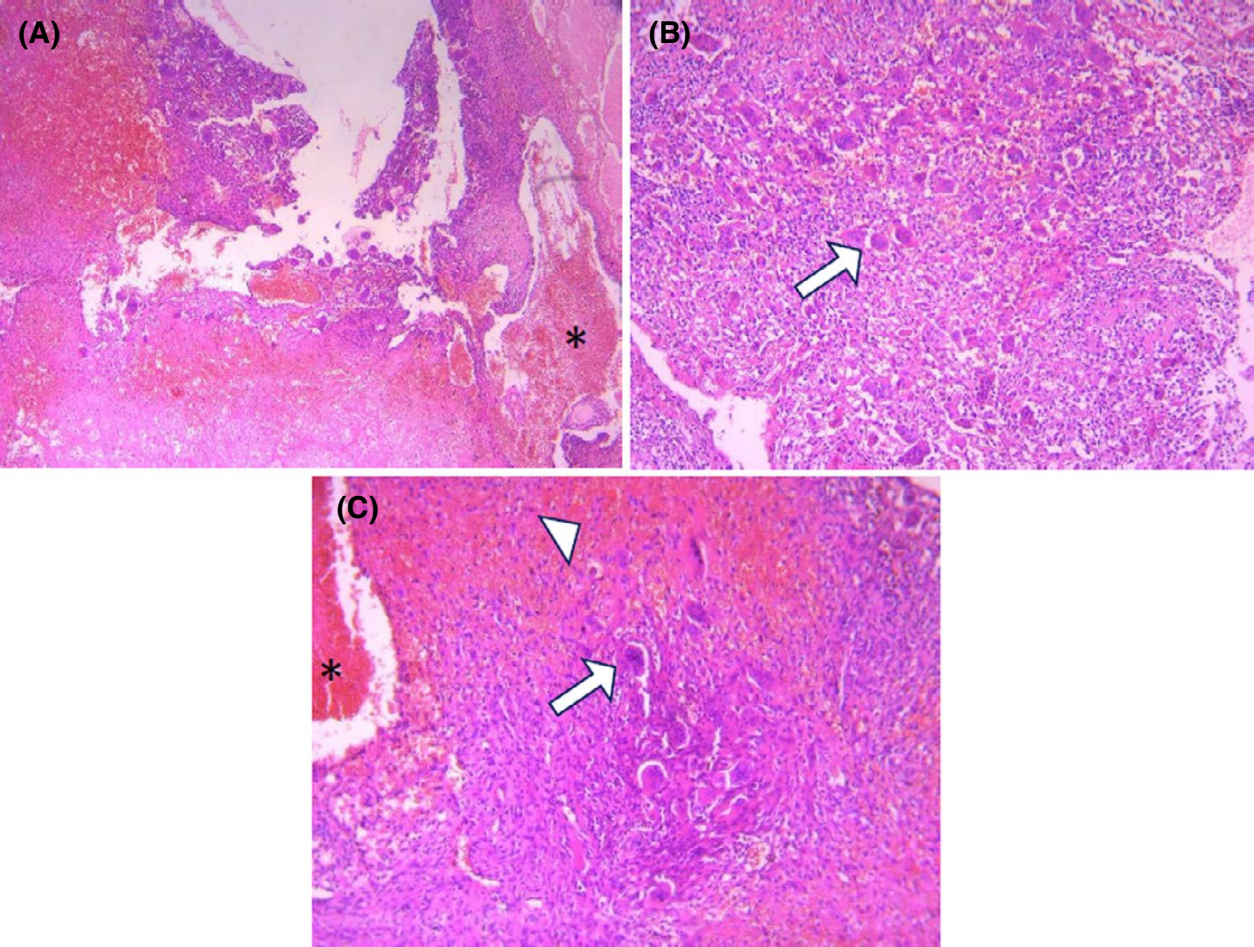
H&E‐stained tissue of resected specimen demonstrating (A) blood‐filled cystic spaces (asterisk sign) ×100, and (B) stromal giant cells (white arrow) in favor of aneurysmal bone cyst ×200. (C) On re‐biopsy, local hemorrhage (white arrowhead), blood‐filled cystic spaces (asterisk sign), and stromal giant cells (white arrow) without anaplastic cells were consistent with aneurysmal bone cyst recurrence ×200

The patient underwent subtotal resection, and pathological examination revealed multiple irregular blood‐filled and empty cystic spaces separated by connective tissue septations containing spindle cells, giant cells, and capillaries with varying amounts of the matrix. Few mitotic figures and fragments of fibroadipose tissue and atrophic skeletal muscle fibers were also detected (Figure 2A, B). Lack of anaplastic stromal cells ruled out the telangiectatic osteosarcoma, and the prominent cystic components ruled out GCTB; Thus, ABC was raised as the final diagnosis. The histological reports were reviewed by another experienced pathologist. Thirty days later, fixation of the spinal column by the metallic screw was performed.

On March 11, 2020, the patient presented with sudden onset of weakness involving all four limbs. Physical examination revealed tenderness over upper cervical vertebrae with spastic quadriparesis (based on the Medical Research Council [MRC], 3/5 in proximal muscles and 4/5 in distal muscles of all extremities). Spinal CT scan showed large heterogeneous and partially hyperdense lesions around the upper cervical spine extending to the prevertebral space and bulging to the nasopharynx. MRI demonstrated cervical canal narrowing. After an incisional biopsy, the diagnosis of ABC recurrence was made, and the patient underwent intralesional curettage of the tumor (Figure 2C). Because of the high risk of severe neurologic sequela and intraoperative bleeding, the patient was not a candidate for surgical resection.

Three days later, the embolization of the vertebral artery and left ascending cervical artery was performed using onyx and coil, with no improvement in quadriparesis after one month. Radiation therapy was the next option done with a total prescribed dose of 30 Gy, delivered 5 days per week at a 1.8 Gy daily dose to the residual tumor; however, the quadriparesis did not improve after 2 weeks.

The case was discussed in a multidisciplinary group involving Oncology, Neurosurgery, Physical Medicine and Rehabilitation, and Hospital Pharmaceutics. It was decided to start denosumab (Xgeva) 120 mg every 4 weeks administered subcutaneously. He received the 1st cycle of denosumab in May 2021. In the laboratory examinations, he had serum urea 23.3 mg/dl, creatinine 0.6 mg/dl, calcium 10.4 mg/dl, and phosphate 4.8 mg/dl.

## 4 OUTCOME AND FOLLOW‐UP

**FIGURE 3:**
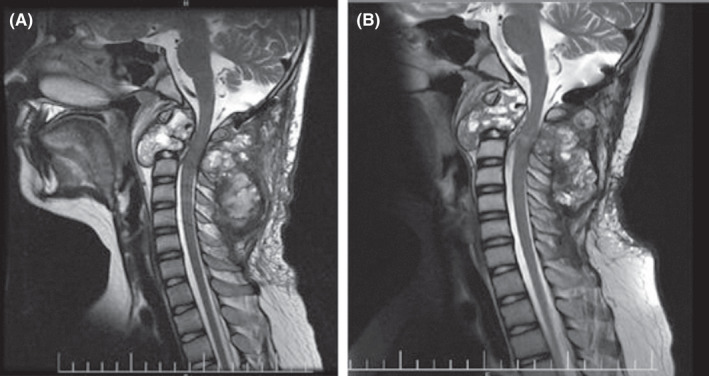
Craniocervical T2W sagittal image (A) before and (B) after six cycles of denosumab administration

He continued to be evaluated at the oncology clinic by physical examination and laboratory testing before each denosumab injection. Calcium and vitamin D supplements were administrated routinely during the treatment. To minimize the risk of osteonecrosis of the jaw, the dental examination was done before treatment, good oral hygiene practices were learned to the patient, and the relevant symptoms were checked during the treatment. Before the 2nd cycle, the neurologic examination revealed a significant improvement in proximal muscle strength (MRC 4/5) with retained distal muscle strength (MRC 4/5) of all extremities. In subsequent cycles, he experienced gradual rehabilitation, and now, after 12 courses, the neurologic symptoms fully recovered (MRC 5/5). After six cycles of denosumab, T2W MRI showed a decrease in fluid–fluid levels and tumor size (Figure 3). The 4th outbreak of COVID‐19 in Iran impeded obtaining a follow‐up MRI.

## 5 DISCUSSION

We presented a patient with ABC—resistant to surgery, selective arterial embolization, and radiotherapy—who responded dramatically to denosumab. ABCs are infrequent (0.14 per 10 people) biologically benign tumors with aggressive behavior and a tendency for local recurrence. It is more common in young women aged <20 years.^1^ ABC can potentially involve any bone in the body, but it usually involves appendicular bones (lower limb >upper limb). Vertebral involvement is only reported in 20% of cases. There are two types of ABC: (i) primary (_~_70%) and (ii) secondary, which occurs in a preexisting bone disorder (e.g., giant cell tumor, osteoblastoma, and fibrous dysplasia).^2^ The most common symptoms of vertebral ABC are pain, swelling, and neurologic dysfunction (motor >sensory). Also, it might be detected incidentally in imaging or secondary to pathological fracture.^1^ The macroscopic view of ABC typically consists of blood‐filled cysts, enclosed in a subperiosteal shell of reactive bone. On the microscopic view, the classic pathological features are (i) blood‐filled multilocular cystic spaces separated by cellular septa containing fibroblasts and giant cells, (ii) exuberant mitotic activity without definite anaplasia, and (iii) micronecrosis‐induced tissue calcification that produces characteristic blue reticulated chondroid‐like material.^1^, ^5^, ^6^

Treatment options consist of surgery (including total or subtotal excision±bone graft, curettage), selective arterial embolization, intralesional injections (calcitonin+steroid, or doxycycline), sclerotherapy, radiotherapy, and denosumab.^2^ In the following section, the evidence for the common treatment options of ABC is summarized. Also, the existing English literature for case study research of denosumab efficacy in ABC is presented in Table 1.

**TABLE 1:** Characteristics of studies applying denosumab in aneurysmal bone cyst (2015–2021)

Study	Skubitz ^15^	Ghermandi^16^	Patel^17^	Kurucu^18^	Kulkarni ^19^	Palmerini^13^	Dürr ^20^	Raux ^14^	Current study
Type	Case report	Case series	Case report	Case series	Case report	Case series	Case series	Case series	Case report
Year	2015	2016	2017	2017	2019	2018	2019	2019	2021
Age/sex	27 years/M	2 patients 42 years/F 16 years/M	16 years/M	9 patients 5M−4F Median age:12.5 years	14 years/F	9 patients 6M−3F Median age:17 years	6 patients 2M−4F Mean age:17 years	5 patients 3M−2F Median age: 8 years	13 years/M
Clinical presentation	Pain	Pain: 2 Neurologic symptom: 1	Pain and movement restriction	Pain: 7 Swelling: 3 Limbing: 3 Pathological fracture: 2	Pain and unsteady gait	Asymptomatic:1 Pain:7 Radiculopathy:1 Paresthesia:1	Majority pain	Pain and neurologic symptoms	Pain and neurologic symptoms
Site	Sacrum	Spine (L5‐S1) Spine (L5)	Spine (C1)	Spine/pelvic	Spine (T5)	Spine/pelvis: 6 Ulna: 1 Tibia: 1 Humerus: 2	Sacrum: 1 Radius: 1 Femur: 1 Talus: 1 Pelvis: 2	Spine: 4 Femur: 1	Spine (C2)
Clinical response	Yes	Yes	Yes	Yes	Yes	Yes	Yes	Yes	Yes
Radiological response	Yes	Yes	Yes	Yes	Yes	Yes	Yes	Yes	Yes
Follow‐up	N/A	33 months and 35 months	12 months	Median:15 months	24 months	Median: 23 months	N/A	Median: 24 months	12 months
Recurrence	N/A	No	No	Radiological recurrence:2 After D/C: 1 During treatment: 1	No	No	After D/C: 2 During treatment: 1	No	No
Adverse event	No	N/A	No	Fatigue: 2 GI toxicity:1 muscle pain:1 Hypocalcemi: 2	N/A	Vomiting: 1	Ca‐rebound abnormality after end of treatment: 1	Hypocalcemia: 2 Hypophosphatemia: 2	No
Dosage	120 mg d1, 8, 15, 28 then every 4 weeks	120 mg d1, 8, 15, 28 then every 40 days	120 mg every 4 weeks	70 mg/m^2^ d1, 8, 15, 28 then monthly	120 mg d1, 8, 15, 28 then every 4 weeks	120 mg d1, 8, 15, 21 then every 4 weeks	120 mg d1, 8, 15, 28 then every 4 weeks	70 mg/m^2^ up to 120 mg weekly for 4 weeks then every 4 weeks	120 mg every 4 weeks
Duration of treatment	1 year	11 cycles, 13 cycles	12 months	Median:12 months or 15 cycles	6 months	Median: 8 cycles	12 months	Median:12 months	12 cycles

### 5.1 Surgery

Complete or en bloc resection has the highest rate of cure and the lowest rate of recurrences. However, it may not be feasible in all cases, and curettage of the lesion with bone grafting might become choice. Recurrence usually occurs in a short interval, commonly seen in younger ages with more extensive lesions.^1^, ^2^

### 5.2 Radiation therapy

The most common hypothesis regarding the radiation effectiveness is small–blood vessel obliteration leading to reduced blood supply.^7^ Because of the risk of secondary malignancy, myelopathy, and deformity of the vertebra, there is a concern to apply radiotherapy as a first‐line option in ABC. However, evidence demonstrates its excellent control rates and safety.^1^, ^7^, ^8^ The German Cooperative Group on Radiotherapy for Benign Diseases recommends fractionated radiation below 30 Gy.^8^

### 5.3 Selective arterial embolization

Selective arterial embolization is a feasible, easily repeatable, and low‐risk option for ABC treatment, especially when there is a high risk of intraoperative bleeding. Approximately 39% recurrence (or persistence) rate has been reported after embolization, but this procedure can be repeated. The possible complications might include ischemic events.^9^

### 5.4 Intralesional injection

Several options are available: (i) Good results have been reported with intralesional injection with calcitonin and methylprednisolone acetate^10^; (ii) intralesional injection of P^32^ has been reported as a viable, safe, and practical option for the management of ABC^11^; and (iii) percutaneous doxycycline injection demonstrated healing response and cortical thickening with a low recurrence rate.^12^

### 5.5 Denosumab

Receptor activator of the nuclear factor‐kappa B ligand (RANKL) is highly expressed in the stroma of ABCs, and the RANK signaling pathway is essential for ABC progression.^2^, ^13^ Given the similar signaling pathway with GCTB and successful results of denosumab in GCTBs, it has been raised as a potential choice in the management of ABC. Denosumab is a fully human monoclonal antibody (IgG2/kappa) binding to RANKL and impedes osteoclast activation. The safety of denosumab has been shown in several clinical trials. Its most significant adverse effects are hypophosphatemia (32%), hypocalcemia (18%), and osteonecrosis of the jaw (1.5%).^4^ Although limited, the published literature is encouraging for denosumab in ABC (Table 1).

Raux et al.^14^ reported the clinical results of denosumab in five children with inoperable ABC (4 in the spine). Denosumab was given 70 mg/m^2^ (up to 120 mg) weekly for 4 weeks and monthly thereafter. After a median follow‐up of 24 months, pain resolved in all and neurologic deficit improved in three patients, with favorable toxicity profile. Imaging showed a decrease in cystic lesions, fluid–fluid levels, contrast enhancement, and bone healing.

These findings align with Palmerino et al.'s^13^ case series of nine older patients (age 14–42 years) with ABC resistance to surgery and embolization. Denosumab was administered on the same schedule, and clinical improvements were detected in all cases with long‐term tumor control. The summary of other case study research is presented in Table 1. In summary, the existing literature addresses the therapeutic benefits of denosumab in the management of patients with ABC in terms of clinical (e.g., pain relief and long‐term tumor control) and radiological (e.g., tumor downstaging and bone healing) aspects. These benefits happen in accordance with a favorable toxicity profile. The most common adverse effects of denosumab in this setting are hypocalcemia and hypophosphatemia.

In conclusion, there is indeed a limited number of studies reporting the clinical results of denosumab in ABC. This case report, along with other case study researches, demonstrated the robust benefits of denosumab in resistant ABC. These encouraging findings can be a clue for further clinical studies to delineate denosumab's efficacy in this setting better.

## CONFLICT OF INTEREST

The authors declared no conflict of interests.

## AUTHOR CONTRIBUTIONS

R.H, M.V, and M.G: drafted and wrote the article and designed the figures. F.T.H: edited and reviewed the article. P.F and AM.A: revised the manuscript critically and provided final suggestions for final preparation.

## ETHICAL APPROVAL

The patient signed informed consent.

## CONSENT

Published with written consent of the patient.

## Data Availability

The data sets used in the current case report are available upon request from the corresponding author.
